# Evaluation of the antibacterial and antifungal properties of oleuropein, olea Europea leaf extract, and thymus vulgaris oil

**DOI:** 10.1186/s12906-024-04596-x

**Published:** 2024-08-09

**Authors:** Fuad Al-Rimawi, Mahmood Sbeih, Mousa Amayreh, Belal Rahhal, Samer Mudalal

**Affiliations:** 1 Chemistry Department, Faculty of Science and Technology, Al-Quds University, P.O. Box 20002, Jerusalem, Palestine; 2 Chemistry Department, Faculty of Applied Sciences, Palestine Technical University-Kadoorie (PTUK), P.O. Box 7, Tulkarm, Palestine; 3 Department of Biomedical Sciences, Faculty of Medicine and Health Sciences, An-Najah National University, P.O. Box 7, Nablus, Palestine; 4 Department of Nutrition and Food Technology, Faculty of Agriculture and Veterinary Medicine, An-Najah National University, P.O. Box 7, Nablus, Palestine

**Keywords:** Oleuropein, Thyme, Antimicrobial, Olive leaf extracts

## Abstract

**Background:**

Although synthetic preservatives and antioxidants may have high antimicrobial and antioxidant activity, they are usually associated with adverse effects on human health. Currently, there is a growing interest in natural antimicrobial and antioxidant agents. This study aimed to evaluate the antimicrobial activity of two medicinal plant extracts and one active compound. Olive leaf extracts (0.2, 0.3, and 0.4% w/v), oleuropein (0.2, 0.4, and 0.6% w/v), thyme oil (0.1%), and oleuropein in combination with thyme oil (0.4% w/v and 0.1% v/v) were used against three bacterial strains (*Escherichia coli*, *Pseudomonas aeruginosa*, and *Staphylococcus aureus*) and two fungal strains (*Candida albicans* and *Aspergillus niger*).

**Results:**

The use of oleuropein resulted in complete antimicrobial activity against *Staphylococcus aureus*, *Pseudomonas aeruginosa*, and *Escherichia coli*. In this context, a reduction of 7 logs was achieved during the storage period (4 weeks). Oleuropein showed no fungal activity at low concentrations (0.2%), but *Aspergillus niger* was reduced by 2.35 logs at higher concentrations (0.6% w/v). Similar antibacterial and antifungal properties were observed for the olive leaf extracts. Oleuropein at a concentration of 0.4 w/v and a mixture of oleuropein and thyme at concentrations of 0.4 and 0.1 (v/v) showed strong antimicrobial activity against the studied microorganisms.

**Conclusion:**

Olive leaf extract, thyme oil, and oleuropein have strong antibacterial and weak antifungal properties. There was a good synergistic effect between oleuropein and thymol.

## Introduction

Medicinal plants and their essential oils have antimicrobial properties [1]. Moreover, they have been investigated as growth-promoting agents for poultry [2, 3]. The use of medicinal plant extracts as alternatives to conventional natural preservatives is growing because they are generally safe for humans and environmentally friendly [4]. Accordingly, interest in the use of phytochemicals as new sources of natural antioxidants and antimicrobial agents is increasing. The use of synthetic antioxidants in the food industry is limited both in terms of application and quantity [5].

Currently, there is a strong debate on the safety of chemical preservatives since they are considered responsible for many carcinogenic and teratogenic properties as well as residual toxicity [6]. Therefore, there is a need to evaluate the efficacy of natural plant extracts to determine their antimicrobial activity. In this context, olive leaf extract is effective against a broad spectrum of pathogenic microorganisms, such as viruses, bacteria, and even parasites. Olive leaf extract can be considered one of the most useful and safe natural antimicrobial herbal extracts discovered to date [7]. Hence, the leaves of *Olea europaea L.* are a typical herbal drug in the Mediterranean region that is widely used in traditional medicine as a vasodilatory, hypotensive, anti-inflammatory, antirheumatic, diuretic, antipyretic, and hypoglycemic agent [8].

Indeed, olive leaves have a large number of active constituents, including oleuropein (the main constituent; 60–90 mg/g) and several types of polyphenolic compounds. Oleuropein is the most studied ingredient. Moreover, 95 different chemicals are present in olive leaves. The content of oleuropein varies from 17 to 23% depending on the farming conditions in which the leaves are harvested [9, 10].

Oleuropein has high antioxidant activity in vitro, comparable to that of a hydrosoluble analog of tocopherol [11]. Oleuropein has shown potent antimicrobial activity against both gram-negative and gram-positive bacteria and against Mycoplasma. The exact mechanism of the antimicrobial activity of oleuropein has not been fully elucidated, although some authors have proposed that it is due to the presence of the ortho-diphenolic system (catechin) [12].

Furthermore, the essential oils of thyme species contain large amounts of thymol, which is a potent antibacterial agent [13] as well as a strong antiseptic and antioxidant [14]. The essential oil of *Thymus vulgaris* contains a mixture of monoterpenes. The two main components of this oil are the natural terpenoid thymol and its phenol isomer [15].

The essential oil of *T. vulgaris* has a high content of oxygenated monoterpenes (56.53%) and a low content of monoterpene hydrocarbons (28.69%), sesquiterpene hydrocarbons (5.04%), and oxygenated sesquiterpenes (1.84%) [16]. The predominant compound among the essential oil components was thymol (51.34%), while the amount of all other constituents of the oil was less than 19%.

There is still a need to investigate the effects of herbal extracts at different concentrations and under different extraction conditions on different strains of microorganisms to maximize their use in the pharmaceutical industry. Moreover, there is a need to evaluate the synergistic effect of these extracts to optimize their use. The aim of this study was to evaluate the antimicrobial activity of olive leaf extract, oleuropein, and thyme oil at different concentrations, either alone or in combination, against three bacterial strains (*Escherichia coli*, *Pseudomonas aeruginosa*, and *Staphylococcus aureus*) and two fungal strains (*Candida albicans* and *Aspergillus niger*).

## Materials and methods

### Collection and preparation of samples

#### Olive and thyme leaves

In November 2017, samples of olive leaves were collected from a field of olive trees in the West Bank, Palestine, which is close to Bethlehem City (latitude 31.70487 and longitude 35.20376). Regarding the thyme leaves, they were gathered in Bethlehem City, West Bank, in April of 2017. Dr. Khalid Sawalha (PhD in Botany, Al-Quds university) is the person who undertook the formal identification of the plant materials used in this study, and assigned voucher number of Pharm-PCT-279 at Al-Quds university herbarium.

The leaves were allowed to dry at ambient temperature (25–30 °C), then they were crushed and sieved using a 120 mm mesh sieve. Until it was extracted using water distillation, the resultant powder was stored at room temperature in the dark. The thyme leaves were processed under the same conditions.

#### Extraction techniques from olive and thyme leaves

The extraction of olive leaves was carried out by water distillation. Approximately 10 g of olive leaf powder was macerated in 100 ml of 80% ethanol (Beit Jala Pharmaceutical Co., Ltd., Bethlehem, Palestine) for 4 h at 40 °C. The extracts were then filtered through a Whatman No. 1 filter (Whatman, UK) to separate the coarse particles from the solution. Then, the filtrate was evaporated at room temperature under vacuum using a rotary evaporator. The concentrated oil extract was stored in a refrigerator at 2–4 °C until use. Permission for the collection of olive and thyme leaves was obtained from the owner of the field.

Thyme oil was extracted from the thyme leaves by steam distillation using water. This method is suitable for producing flower blossoms and finely powdered plant material. The distillation temperature was approximately 100 °C at atmospheric pressure. Approximately 55 g of dried thyme was placed in the steam distillation apparatus. The leaves of the thyme plants were immersed in water and boiled. Then, the instrument was turned on, and boiling water was used to extract the essential oils from the sacs of the leaves. The steam containing the essential oil was passed through a cooling system for condensation. Finally, the oil was separated from the water. The antibacterial activity of hydrosol (a mixture of oil and water) was tested.

### Antimicrobial activity test

This procedure was used to evaluate the antimicrobial efficacy of the extracts. A selected agar medium was used for the cultivation of the organisms under investigation and for the rigorous growth of each stock culture. The recommended media used were soybean casein digest agar (SCDA)/broth (SCDB) for *Escherichia coli*, *Pseudomonas aeruginosa*, and *Staphylococcus aureus*. In addition, Sabouraud dextrose agar (SDA)/broth (SDB) were used to *Candida albicans* and *Aspergillus niger*. If necessary, an appropriate inactivator (neutralizer) was added to the broth and/or agar media for the specific antimicrobial properties of the product. A stock culture of each tested microorganism was prepared using sterile saline TS to obtain a microbial count of about 1 × 10^8^ CFU/ml. These cultures (two fungal species (*Candida albicans* (ATCC # 10,231) and *Aspergillus niger* (ATCC # 16,404)) and three bacterial species (*Escherichia coli* (ATCC # 8739), *Pseudomonas aeruginosa* (ATCC # 9027), and *Staphylococcus aureus* (ATCC # 6538)) were used to assess the suitability of selected media to support the growth of tested microorganisms using growth promotion test (Table 1).

**Table 1:** Growth promotion test for selected microbes in soybean casein digest and Sabouraud dextrose agar and broth

Media	Sabouraud dextrose agar		Soybean casein digest agar		Sabouraud broth		Soybean casein digest broth	
Parameters								
Appearance	Light beige, free flowing and homogenous. **Result: pass**		Light beige, free flowing and homogenous. **Result: pass**		Light beige, free flowing and homogenous. **Result: pass**		Light beige, free flowing and homogenous. Result: pass	
Solubility/color of solution	6.5% solution, soluble in distilled water or deionized water on boiling. Solution is light to medium amber without precipitate **Result: pass**		4% solution, soluble in distilled water or deionized water on boiling. Solution is light to medium amber slightly opalescent. **Result: pass**		6.5% solution, soluble in distilled water or deionized water on boiling. Solution is light to medium amber without precipitate **Result: pass**		3% solution, soluble in distilled water or deionized water on boiling. Solution is light amber, clear Result: pass	
Appearance of prepared medium	light to medium amber, slightly opalescent without precipitate **Result: pass**		Light amber, slightly opalescent with no significant precipitate. **Result: pass**		Very light amber, slightly opalescent without precipitate **Result: pass**		Light amber, clear Result: pass	
pH	5.6 *±* 0.2 **Result: pass**		7.3 *±* 0.2 **Result: pass**		5.6 *±* 0.2 **Result: pass**		7.3 *±* 0.2 Result: pass	
Microorganism^2^/Incubation time	*C. albicans*/3 days	*A. Niger/*5 days	*S. aureus*/18–24 h	*E. coli*/18–24 h	*A. Niger/*18–24 h	*C. albicans*/18–24 h	*S. aureus*/18–24 h	*E. coli*/18–24 h
10^1^ inoculums (CFU/ml)^1^	> 10	> 10	> 10	> 10	Turbid	Turbid	Turbid	Turbid
10^2^ inoculums (CFU/ml)	> 100	> 100	> 100	> 100	Turbid	Turbid	Turbid	Turbid
10^3^ inoculums (CFU/ml)	> 1000	> 1000	> 1000	> 1000	Turbid	Turbid	Turbid	Turbid

^1^ CFU: Colony Forming Unit

The results are considered pass when they comply with the manufacturer’s specifications for the media

^2^ The results showed that media can support the growth of microbes under investigation

The surface of a suitable volume of solid agar medium was inoculated from a recently revived stock culture of each of the indicated microorganisms. The culture conditions for the inoculum preparations are described in Table 2. A similar procedure, but without the addition of extracts, was used for the control test

**Table 2:** Incubation conditions for different cultures of bacterial and fungal strains

Organism	Suitable medium	Incubation temperature	Inoculum incubation time	Microbial recovery incubation time
*Escherichia coli* ATCC No. 8739	SCDB, SCDA	32.5 + 2.5 °C	18–24 h	3–5 days
*Pseudomonas aeruginosa* ATCC No. 9027	SCDB, SCDA	32.5 + 2.5 °C	18–24 h	3–5 days
*Staphylococcus aureus* ATCC No. 6538	SCDB, SCDA	32.5 + 2.5 °C	18–24 h	3–5 days
*Candida albicans* ATCC No. 10,231	(SDA)/ (SDB)	22.5 + 2.5 °C	44–52 h	3–5 days
*Aspergillus niger* ATCC No. 16,404	(SDA)/ (SDB)	22.5 + 2.5 °C	6–10 days	3–7 days

Different broths of soybean casein digest containing olive leaf extracts (0.2, 0.3, and 0.4% w/v), oleuropein (0.2, 0.4, and 0.6% w/v), thyme oil (0.1%), and oleuropein in combination with thyme oil (0.4% w/v and 0.1% v/v) were prepared for evaluating the antibacterial activity. For each type of broth, an inoculum of selected bacterial species (about 1 × 10^8^ CFU/ml) was added to each broth. In the same way, the antifungal activity was evaluated using Sabouraud dextrose broth. The final microbial load was evaluated using the membrane filtration method. 10 ml of each type of broth was filtrated through a 0.45-micrometer membrane filter about 50 mm in diameter. For bacterial evaluation, a membrane filter was placed on the surface of solidified SCDA plates and incubated at 30–35 °C for 48–72 h. Similarly, for fungal evaluation, a membrane filter was placed on the surface of solidified SDA plates and incubated at 20–25 °C for 5–7 days. Several dilutions (10^− 3^-10^− 6^) were prepared for each sample to obtain plates containing between 30 and 100 CFU.

## Results

The antimicrobial activity of oleuropein (0.2% w/v) against *Staphylococcus aureus*,* Pseudomonas aeruginosa*, *Escherichia coli*, *Candida albicans*, and *Aspergillus niger* compared with that of the control is shown in Table 3. Oleuropein (0.2%) exhibited complete antimicrobial activity against *Staphylococcus aureus*, *Pseudomonas aeruginosa* and *Escherichia coli* during the fourth week of storage. The initial microbial load for the three tested bacteria decreased by approximately 7 logs. Oleuropein at a concentration of 0.2% showed no fungal activity against *Candida albicans* or *Aspergillus niger*. *Staphylococcus aureus* was more susceptible (complete inhibition in the first week) to oleuropein than were *Pseudomonas aeruginosa* and *Escherichia coli* (complete inhibition in the fourth week). In the control treatment, an increase in microbiological counts was observed at the beginning of treatment, followed by a decrease in counts.

**Table 3:** Antimicrobial activity of oleuropein (0.2% w/v) against *Staphylococcus aureus*, *Pseudomonas aeruginosa*, *Escherichia coli*, *Candida albicans*, and *aspergillus Niger* compared to the control

Microorganism Species	^1^ATCC NO.	Storage Time (Days)	Mean (Log cfu/ml)	
			Treatment	Control
*S. aureus*	6538	0	7.34	6.71
		7	< 1*	9.24
		14	< 1	8.92
		28	< 1	7.86
*P. aeruginosa*	9027	0	6.93	6.23
		7	6.35	9.10
		14	3.73	8.64
		28	< 1	7.57
*E. coli*	8739	0	7.38	6.58
		7	4.80	9.00
		14	3.73	8.78
		28	< 1	7.98
*C. albicans*	10,231	0	7.41	6.25
		7	7.43	8.16
		14	7.08	8.00
		28	6.20	6.95
A. *niger*	16,404	0	7.33	5.76
		7	5.91	4.87
		14	5.55	4.82
		28	5.60	3.45

^1^ ATCC: American Type Culture Collection

* If there was no growth, the result was expressed as < 1 log (cfu/ml) for a negative result

The antimicrobial activity of oleuropein (0.4% w/v) against *Staphylococcus aureus*, *Pseudomonas aeruginosa*, *Escherichia coli*, *Candida albicans*, and *Aspergillus niger* compared to control is shown in Table 4. Our study showed that 0.4% oleuropein exhibited complete antimicrobial activity against *Staphylococcus aureus*, *Pseudomonas aeruginosa*, and *Escherichia coli* in the second week of storage. A reduction of 7 log cycles in the initial microbial load was observed for the three tested bacteria. In general, *Candida albicans* and *Aspergillus niger* were not affected by the addition of 0.4% oleuropein. Exceptionally, the number of *Aspergillus niger* was reduced by 1.56 log cycles at the end of storage compared with the initial count. A similar pattern was observed for bacterial susceptibility to 0.40% oleuropein, where *Staphylococcus aureus* was more sensitive (complete inhibition in the first week) to oleuropein than were *Pseudomonas aeruginosa* and *Escherichia coli* (complete inhibition in the second week). Increasing the concentration of oleuropein from 0.2 to 0.4% resulted in a one-week reduction in the time required to achieve complete inhibition of *Pseudomonas aeruginosa* and *Escherichia coli*. In the control treatment, a similar pattern was observed in microbial growth, where at the beginning, there was an increase in microbiological counts followed by a reduction in the count.

**Table 4:** Antimicrobial activity of oleuropein (0.4% w/v) against *Staphylococcus aureus*, *Pseudomonas aeruginosa*, *Escherichia coli*, *Candida albicans*, and *aspergillus Niger* compared to the control

Microorganism Species	^1^ATCC NO.	Storage Time (Days)	Mean (Log cfu/ml)	
			Treatment	Control
*S. aureus*	6538	0	7.33	6.71
		7	< 1*	9.24
		14	< 1	8.92
		28	< 1	7.86
*P. aeruginosa*	9027	0	6.77	6.23
		7	4.53	9.10
		14	< 1	8.64
		28	< 1	7.57
*E. coli*	8739	0 7	7.27 5.37	6.58 9.00
		14	< 1	8.78
		28	< 1	7.98
*C. albicans*	10,231	0	6.41	6.25
		7	6.04	8.16
		14	6.88	8.00
		28	6.56	6.95
A. *niger*	16,404	0	5.80	5.76
		7	5.23	4.87
		14	5.29	4.82
		28	4.24	3.45

^1^ ATCC: American Type Culture Collection

* If there was no growth, the result was expressed as < 1 log (cfu/ml) for a negative result

The antimicrobial activity of oleuropein (0.6% w/v) against *Staphylococcus aureus*, *Pseudomonas aeruginosa*, *Escherichia coli*, *Candida albicans*, and *Aspergillus niger* compared with that of the control is shown in Table 5. Our study showed that 0.6% oleuropein had complete antimicrobial activity against *Staphylococcus aureus*, *Pseudomonas aeruginosa*, and *Escherichia coli* at the end of the second week of storage. A reduction of 7 log cycles in the initial microbial load was observed for the three tested bacteria.

In general, *Candida albicans* and *Aspergillus niger* were not affected by the addition of 0.6% oleuropein. Exceptionally, the number of *Aspergillus niger* was reduced by 2.35 log cycles at the end of storage compared to the original number. A similar pattern was observed for bacterial sensitivity to 0.40% oleuropein, with *Staphylococcus aureus* being more sensitive (complete inhibition in the first week) to oleuropein than *Pseudomonas aeruginosa* and *Escherichia coli* (complete inhibition in the third week).

**Table 5:** Antimicrobial activity of oleuropein (0.6% w/v) against *Staphylococcus aureus*, *Pseudomonas aeruginosa*, *Escherichia coli*, *Candida albicans*, and *aspergillus Niger* compared to the control

Microorganism Species	^1^ATCC NO.	Storage Time (Days)	Mean (Log cfu/ml)	
			Treatment	Control
*S. aureus*	6538	0	6.84	06.71
		7	< 1	10.24
		14	< 1	08.92
		28	< 1	07.86
*P. aeruginosa*	9027	0	5.46	06.23
		7	3.85	09.10
		14	< 1	08.64
		28	< 1	07.57
*E. coli*	8739	0	6.73	06.08
		7	5.04	09.00
		14	< 1	08.78
		28	< 1	07.98
*C. albicans*	10,231	0	6.74	06.01
		7	6.28	08.16
		14	6.13	08.00
		28	4.79	08.10
A. *niger*	16,404	0	5.48	05.76
		7	3.58	04.95
		14	3.25	05.82
		28	3.13	04.45

^1^ ATCC: American Type Culture Collection

* If there was no growth, the result was expressed as < 1 log (cfu/ml) for a negative result

The antimicrobial activity of the olive leaf extracts (0.2, 0.3, and 0.4% w/v) against *Staphylococcus aureus*, *Pseudomonas aeruginosa*, *Escherichia coli*, *Candida albicans*, and *Aspergillus niger* compared to that of the control is shown in Tables 6 and 7, and 8. The use of 0.2% olive leaf extracts resulted in complete inhibition of the microbial growth *of Staphylococcus aureus*, *Pseudomonas aeruginosa*, and *Escherichia coli* during the fourth week of storage. *Staphylococcus aureus* was more sensitive (complete inhibition in the first week) to olive leaf extracts than were *Pseudomonas aeruginosa* and *Escherichia coli* (complete inhibition in the fourth week). The addition of 0.2% olive leaf extracts had no inhibitory effect on *Candida albicans*, while *Aspergillus niger* had a moderate effect, with a 1.81 log reduction in the count at week four.

**Table 6:** Antimicrobial activity of OLE (0.2% w/v) against Staphylococcus aureus, Pseudomonas aeruginosa, Escherichia coli, Candida albicans, and aspergillus Niger compared to the control

Microorganism Species	^1^ATCC NO.	Storage Time (Days)	Mean (Log cfu/ml)	
			Treatment	Control
*S. aureus*	6538	0	7.40	7.71
		7	< 1	9.24
		14	< 1	8.92
		28	< 1	7.36
*P. aeruginosa*	9027	0 7	7.06 5.16	5.73 9.10
		14	2.97	8.64
		28	< 1	7.57
*E. coli*	8739	0	7.41	6.58
		7	6.13	8.00
		14	3.29	8.78
		28	< 1	7.98
*C. albicans*	10,231	0	6.79	6.25
		7	7.44	8.16
		14	7.23	8.00
		28	6.94	8.10
A. *Niger*	16,404	0	6.38	5.76
		7	5.74	4.95
		14	5.75	4.89
		28	4.57	4.82

^1^ ATCC: American Type Culture Collection

* If there was no growth, the result was expressed as < 1 log (cfu/ml) for a negative result

Compared to 0.2% olive leaf extracts, 0.3% olive leaf extracts exhibited similar inhibitory effects against *Staphylococcus aureus*,* Pseudomonas aeruginosa*, and *Escherichia coli* in the fourth week of storage (Table 7). *Staphylococcus aureus* was more sensitive (complete inhibition in the first week) to olive leaf extracts than were *Pseudomonas aeruginosa* (complete inhibition in the third week) and *Escherichia coli* (complete inhibition in the fourth week). Increasing the concentration of olive leaf extract from 0.2 to 0.3% reduced the time required for complete inhibition of *Pseudomonas aeruginosa* growth by one week. Increasing the concentration of olive leaf extracts to 0.3% had no effect on the growth of *Candida albicans*, while the final concentration of *Aspergillus niger* decreased by 1.56 log.

**Table 7:** Antimicrobial activity of OLE (0.3% w/v) against Staphylococcus aureus, Pseudomonas aeruginosa, Escherichia coli, Candida albicans, and aspergillus Niger compared to the control

Microorganism Species	^1^ATCC NO.	Storage Time (Days)	Mean (Log cfu/ml)	
			Treatment	Control
*S. aureus*	6538	0	7.17	6.71
		7	< 1	9.24
		14	< 1	8.92
		28	< 1	7.86
*P. aeruginosa*	9027	0	6.73	6.23
		7	5.88	9.10
		14	< 1	8.64
		28	< 1	7.57
*E. coli*	8739	0	7.12	7.58
		7	5.95	9.00
		14	3.13	8.78
		28	< 1	8.48
*C. albicans*	10,231	0	7.06	7.06
		7	7.16	8.16
		14	7.08	9.00
		28	6.74	9.10
A. *niger*	16,404	0	6.09	5.76
		7	5.67	4.95
		14	5.65	4.89
		28	4.53	4.82

^1^ ATCC: American Type Culture Collection

* If no growth occurred, the result is expressed as < 1 log (cfu/ml) for a negative result

Increasing the concentration of olive leaf extracts to 0.4% resulted in complete inhibition of *Staphylococcus aureus* growth in the first week, while *Pseudomonas aeruginosa* and *Escherichia coli* were completely inhibited in the second week of storage (Table 8). *Staphylococcus aureus* was more sensitive to olive leaf extracts than were *Pseudomonas aeruginosa* and *Escherichia coli*. The time needed to achieve complete inhibition of *Escherichia coli* growth was reduced by one week. A similar pattern in microbial growth was observed with the control treatment, with an initial increase in microbiological counts followed by a reduction in counts.

A slight antifungal effect was observed when the concentration of olive leaf extract was increased from 0.2 to 0.4%. The final counts of *Candida albicans* and *Aspergillus niger* decreased by 1.09 log and 2.37 log, respectively.

**Table 8:** Antimicrobial activity of OLE (0.4% w/v) against Staphylococcus aureus, Pseudomonas aeruginosa, Escherichia coli, Candida albicans, and aspergillus Niger compared to the control

Microorganism Species	^1^ATCC NO.	Storage Time (Days)	Mean (Log cfu/ml)	
			Treatment	Control
*S. aureus*	6538	0 7	6.90 < 1	6.71 9.24
		14	< 1	8.92
		28	< 1	7.86
*P. aeruginosa*	9027	0	5.43	6.23
		7	3.63	9.10
		14	< 1	8.64
		28	< 1	7.57
*E. coli*	8739	0	6.51	6.58
		7	4.90	9.00
		14	< 1	8.78
		28	< 1	7.98
*C. albicans*	10,231	0	5.71	6.25
		7	7.66	8.16
		14	5.65	8.00
		28	4.62	6.95
A. *niger*	16,404	0	5.27	5.76
		7	3.51	4.87
		14	3.18	4.82
		28	2.90	3.45

^1^ ATCC: American Type Culture Collection

* If there was no growth, the result was expressed as < 1 log (cfu/ml) for a negative result

The antimicrobial activity of 0.1 w/v thyme oil against *Staphylococcus aureus*, *Pseudomonas aeruginosa*, *Escherichia coli*, *Candida albicans*, and *Aspergillus niger* compared with that of the control is shown in Table 9. Thyme oil (0.1% w/v) showed complete antimicrobial activity against *Staphylococcus aureus*, *Pseudomonas aeruginosa*, and *Escherichia coli* during the second week of storage. In the control, a similar pattern was observed for microbial growth, with an increase in microbiological counts at baseline, followed by a reduction in counts. Thyme oil (0.1% w/v) had no antifungal activity against *Candida albicans* or *Aspergillus niger.*

**Table 9:** Antimicrobial activity of thyme oil (0.1% w/v) against Staphylococcus aureus, Pseudomonas aeruginosa, Escherichia coli, Candida albicans, and aspergillus Niger compared to the control

Microorganism Species	^1^ATCC NO.	Storage Time (Days)	Mean (Log cfu/ml)	
			Treatment	Control
*S. aureus*	6538	0	6.70	6.71
		7	4.81	9.24
		14	< 1	8.92
		28	< 1	7.86
*P. aeruginosa*	9027	0	5.41	6.23
		7	3.72	9.10
		14	< 1	8.64
		28	< 1	7.57
*E. coli*	8739	0	6.61	6.58
		7	5.12	9.00
		14	< 1	8.78
		28	< 1	7.98
*C. albicans*	10,231	0	6.43	6.25
		7	6.45	8.16
		14	6.87	8.00
		28	6.13	6.95
A. *Niger*	16,404	0	6.93	5.76
		7	6.61	4.87
		14	6.42	4.82
		28	6.35	3.45

^1^ ATCC: American Type Culture Collection

* If there was no growth, the result was expressed as < 1 log (cfu/ml) for a negative result

The antimicrobial activity of thyme oil (0.1% w/v) and oleuropein (0.4% w/v) against *Staphylococcus aureus*, *Pseudomonas aeruginosa*, *Escherichia coli*, *Candida albicans*, and *Aspergillus niger* compared with the control is shown in Table 10. Thyme oil (0.1% w/v) combined with oleuropein (0.4% w/v) showed complete antimicrobial activity against *Staphylococcus aureus* (in the first week), *Pseudomonas aeruginosa* (in the second week), and *Escherichia coli* (in the second week). Thyme oil (0.1% w/v) with oleuropein (0.4% w/v) had mild antifungal activity against *Candida albicans* and *Aspergillus niger*, with reductions in baseline counts of 2.69 log and 2.25 log, respectively.

**Table 10:** Antimicrobial activity of oleuropein (0.4% w/v) and thyme oil (0.1% v/v) against *Staphylococcus aureus*,* Pseudomonas aeruginosa*,* Escherichia coli*,* Candida albicans*, and *aspergillus Niger* compared to the control

Microorganism Species	^1^ATCC NO.	Storage Time (Days)	Mean (Log cfu/ml)	
			Treatment	Control
*S. aureus*	6538	0	6.79	6.71
		7	< 1	9.24
		14	< 1	8.92
		28	< 1	7.86
*P. aeruginosa*	9027	0	5.53	6.23
		7	3.51	9.10
		14	< 1	8.64
		28	< 1	7.57
*E. coli*	8739	0	6.59	6.58
		7	5.19	9.00
		14	< 1	8.78
		28	< 1	7.98
*C. albicans*	10,231	0	7.10	6.25
		7	5.97	8.16
		14	5.89	8.00
		28	4.41	6.95
A. *niger*	16,404	0	5.12	5.76
		7	3.39	4.87
		14	3.07	4.82
		28	2.87	3.45

^1^ ATCC: American Type Culture Collection

* If there was no growth, the result was expressed as < 1 log (cfu/ml) for a negative result

## Discussion

Overall, *Staphylococcus aureus* was more sensitive (complete inhibition in the first week) to oleuropein (at all concentrations: 0.2, 0.4, and 0.6%) than were *Pseudomonas aeruginosa* and *Escherichia coli* (complete inhibition in the third or fourth week). Increasing the concentration of oleuropein from 0.4 to 0.6% had no effect on the time required to achieve complete inhibition of *Pseudomonas aeruginosa* and *Escherichia coli*. In this regard, it was found that oleuropein had potent antimicrobial activity against both gram-negative and gram-positive bacteria. The mechanism of antimicrobial activity of oleuropein can be attributed to the presence of the ortho-diphenolic system. In this context, it has been suggested that oleuropein may interfere with the production pathways of some amino acids that promote the growth of certain microorganisms. Moreover, oleuropein may stimulate phagocytosis as a response of the immune system to microbes [12].

Our study showed that oleuropein is more effective to gram-positive bacteria than to gram-negative bacteria. This result was in agreement with previous studies. In this context, it was found that oleuropein had antimicrobial activity against *Staphylococcus aureus*,* Salmonella typhimurium*,* Escherichia coli*,* Klebsiella pneumonia*, and *Bacillus cereus* [17]. Similar results were observed by Al-Rimawi et al. [18]. Our findings showed that oleuropein had very slight effect on investigated fungi (in particular, *Candida albican and A. niger*). This result was not in agreement with Zorić and Kosalec [19].

Moreover, Salma [20] showed that OLE (olive oil extract) was effective against two gram-positive strains (*S. aureus* and *B. cereus*). Dermatophytes were inhibited after three days by 1.25% (w/v) OLE, while the growth of *Candida albicans* was inhibited after 24 h in the presence of 15% (w/v) OLE [21]. Several researchers have shown that olive leaf extract has antibacterial and antifungal properties [21–23].

It is interesting to compare oleuropein with OLE in terms of antimicrobial activity. OLE is clearly more effective than oleuropein. This may be due to additional active compounds (e.g., polyphenols and flavonoids) in OLE that may have such activity or act synergistically. Olive leaves contain a large number of functional compounds, such as oleuropein (60–90 mg/g), hydroxytyrosol, tyrosol, elenolic acid derivatives, caffeic acid, oleuropein, verbascoside, rutin, luteolin 7-O-glucoside, luteolin 4-O-glucoside, apigenin-7-O-rutinoside, and apigenin 7-O-glucoside [9]. Several functional compounds, in addition to oleuropein, work in a synergistic way to inhibit the growth of microorganisms [10].

The aqueous extract of olive leaves showed good antimicrobial activity and the highest inhibitory activity against *Salmonella typhi* [24]. The efficacy of OLE may not be due to one main active ingredient but to the action of different ingredients originally present in the plant [25].

The efficacy of OLE against fungi strongly depends on the type of solvent used (water, acetone, methanol, or ethyl acetate) [26]. The results of a previous publication showed that the aqueous extract had the most prominent activity, while diethylether extracts of olive leaves showed low antifungal activity [26].

The efficacy of olive leaf aqueous extracts against selected gram-positive microorganisms (*Bacillus cereus*, *B. subtilis*, and *Staphylococcus aureus)*, gram-negative bacteria (*Pseudomonas aeruginosa*,* Escherichia coli*,* and Klebsiella pneumoniae*), and fungi (*Candida albicans* and *Cryptococcus neoformans*) has also been studied [27]. The results of this study showed that OLE exhibited unusual antibacterial and antifungal activity at low concentrations. The conclusion from this study was that olive leaf extracts and oleuropein have potential activity against microbes.

Increasing the concentration of chloroform olive leaf extract from 0.6 to 3.6 mg has great potential in antimicrobial food packaging to reduce the growth of bacteria after processing [28]. Furthermore, some researchers studied the antibacterial and antifungal properties of olive leaf ethyl acetate acetone extracts (10, 15, 20, 30, and 50 mg/ml) using the agar disk diffusion method, and the study showed a significant effect on the growth of selected microorganisms (*Staphylococcus aureus*, *Enterococcus faecalis*, *Bacillus cereus*, and *Escherichia coli*) [29]. Similar results using different concentrations of OLE and different microorganisms have been obtained in other published works [30, 31].

Olive leaf extract (0.31–0.78%) exhibited antimicrobial activity against *Campylobacter jejuni*,* Helicobacter pylori*, and *Staphylococcus aureus* (including methicillin-resistant *S. aureus* (MRSA)) [30]. A concentration of 40% (w/v) olive leaf extract was necessary to affect the growth of *C. albicans*, while 0.6% (w/v) olive leaf extract was able to completely destroy *Escherichia coli* [21].

The antimicrobial activity of olive leaf extracts depends on many factors, such as the olive cultivar, extraction method (Soxhlet and microwave-assisted extraction, MAE), extraction time, and type of solvent. All these factors affect the total phenolic content, antioxidant activity, phenolic profile of the extracts, and content of oleuropein, which is the predominant compound in all extracts [32, 33]. OLE at a concentration of 62.5 mg/ml completely inhibited *Listeria monocytogenes*, *Escherichia coli O157:H7*, and *Salmonella enteritidis* [33].

According to another previous study, the lowest minimum inhibitory concentration (MIC) was 315 µg/ml, and the minimum bactericidal concentration (MBC) was 2500 µg/ml OLE for *S. aureus* when acetone and methanol were used as solvents. On the other hand, the lowest MIC was 625 µg/ml, and the lowest MBC was 5000 µg/ml for *E. coli* when water and methanol were used as solvents and microwave-assisted extraction was used, respectively [34].

The antimicrobial properties of olive leaf extracts against some yeasts were investigated using different solvent extracts, such as water, ethanol, acetone, and ethyl acetate [35]. The study showed that all solvents except water had minimum inhibitory concentrations in the range of 10–28 µg/ml and minimum fungicidal concentrations in the range of 20–48 µg/ml for all studied yeasts. Previous studies indicated that the aqueous extract of olive leaves had no antibacterial effect, while the acetone extract showed inhibitory effects against *Salmonella enteritidis*, *Bacillus cereus*, *Klebsiella pneumoniae*, *Escherichia coli*, *Enterococcus faecalis*,* Streptococcus thermophilus*, and *Lactobacillus bulgaricus* [36, 37]. Furthermore, it was found that there were differences in the distribution and content of total phenolic compounds in olive leaf extracts obtained from different olive cultivars [38].

In our study, the antifungal properties of olive leaf extracts against *Candida albicans* PTCC-5027 were investigated. Fresh olive leaf extracts were prepared with distilled water in a Soxhlet apparatus. The antifungal activity of the extract was analyzed by measuring the minimum inhibitory concentration (MIC) and MFC using the microdilution test and the disc diffusion assay.

Aqueous olive leaf extracts (with a minimum fungicidal concentration of 48 mg/ml) showed antifungal effects against *Candida albicans* [39]. Giacometti et al. [40] revealed that olive leaf extracts obtained by ultrasound-assisted extraction had greater antioxidant and antimicrobial activities against common foodborne pathogens than those obtained by conventional extraction. It was found that 0.625 mg/ml of olive oil polyphenol extract resulted in complete growth inhibition of *Salmonella typhimurium* and *S. aureus* [41].

Our study showed that thymol had strong antibacterial activity against tested bacterial species. These results were in agreement with previous studies. In this context, it was found that thymol extracted from thyme species has antibacterial activity [13] and strong antiseptic and antioxidant activity [14]. The essential oil of *T. vulgaris* has a high content of oxygenated monoterpenes and a low content of monoterpene hydrocarbons, according to previous reports [16, 42]. They found that thyme oil had a significant effect on some studied microorganisms.

In other study, thyme oil showed antibacterial activity against *Staphylococcus aureus*,* Escherichia coli*,* Bacillus cereus*,* Salmonella enteritidis*,* Salmonella typhimurium*, and methicillin-resistant *Staphylococcus aureus* [43]. Moreover, the effects of the essential oils of thyme and oregano on 43 microorganisms (including 26 bacteria, 14 fungal species, and 3 types of yeasts) were evaluated, and revealed that the minimum inhibitory concentrations for bacterial strains were in the range of 7.8–500 µg/ml [44]. In addition, another report showed that there was potential synergy between thymol-oregano essential oils and carvacrol in terms of antioxidant and antibacterial properties [45].

## Conclusions

The time needed to achieve complete inhibition of the growth was varied according to the tested microorganisms. *Staphylococcus aureus* was more sensitive to oleuropein than *Pseudomonas aeruginosa* and *Escherichia coli* (complete inhibition in the first week versus complete inhibition in the fourth week), respectively. The study showed that increasing the concentration of oleuropein resulted in a reduction in the time needed to achieve complete inhibition of the growth of tested bacterial species, while this pattern was not observed in tested fungal species.

Similarly, *Staphylococcus aureus* showed higher sensitivity toward olive leaf extract than *Pseudomonas aeruginosa* and *Escherichia coli* regarding the time needed to achieve complete inhibition of the growth of the tested microorganisms. Olive leaf extract exhibited mild antifungal activity against *Aspergillus niger* and no antifungal activity against *Candida albicans*. Similar to oleuropein, it was observed that increasing the concentration of olive leaf extract reduced the time needed to achieve complete inhibition for the growth of the tested bacterial species. Olive leaf extract resulted in mild reduction in the fungal count, and this effect is considered as low importance from a technical perspective. Thyme oil showed strong antimicrobial activity at low concentration against *Staphylococcus aureus*, *Pseudomonas aeruginosa*, and *Escherichia coli* while there was no antifungal activity. Overall results indicated that oleuropein, olive leaf extract, and thymol had strong antibacterial activity and weak or slight antifungal activity, regardless of the concentration. There was synergy in antibacterial activity between oleuropein and thyme. Further investigations are needed to optimize the synergy of tested materials to improve their antifungal activity.

## Data Availability

The datasets used and/or analyzed during the current study available from the corresponding author on reasonable request.

## Abbreviations

RH: Relative humidity
HPLC: High performance liquid chromatography
ATCC: American Type Culture Collection
O.D.: Optical density
CFU: Colony-forming units
SCDM: Soyabean Casein Digest Medium
CDSLP: Casein Digest- soy lecithin poly sorbate
FLM: Fluid Lactose Medium
SCDA: Soyabean Casein Digest Agar
TABC: Total Aerobic Bacterial Count
TYMC: Total Combined Yeasts and Molds Count
ppm: Parts per million

## References

[1] Alwafa RA, Mudalal S, Mauriello G. Origanum syriacum L. (Za’atar), from raw to Go: a review. Plants. 2021;10:1001. 10.3390/plants10051001 PMC8156404 34067806

[2] Zaazaa A, Mudalal S, Alzuheir I, Samara M, Jalboush N, Fayyad A, Petracci M. The impact of Thyme and Oregano essential oils dietary supplementation on Broiler Health, growth performance, and prevalence of growth-related breast muscle abnormalities. Animals. 2022;12:3065. 36359189 10.3390/ani12213065 PMC9653697

[3] Mudalal S, Zaazaa A, Omar JA. Effects of medicinal plants extract with antibiotic free diets on broilers growth performance and incidence of muscles abnormalities. Braz J Poult Sci. 2021;23:001–8. 10.1590/1806-9061-2020-1342

[4] Thangavelu R, Sundararaju P, Sathiamoorthy S. Management of anthracnose disease of banana caused by Colletotrichum musae using plant extracts. J Hortic Sci Biotechnol. 2004;79:664–68. 10.1080/14620316.2004.11511823

[5] Alali FQ, Tawaha K, El-Elimat T, Syouf M, El-Fayad M, Abulaila K, Nielsen SJ, Wheaton WD, Falkinham JO, Oberlies NH. Antioxidant activity and total phenolic content of aqueous and methanolic extracts of Jordanian plants. Nat Prod Res. 2017;21:1121–31. 10.1080/14786410701590285 17852749

[6] Moreira MR, Ponce AG, del Valle CE, Roura SI. Inhibitory parameters of essential oils to reduce a Foodborne Pathogen. LWT Food Sci Technol. 2005;38:565–70. 10.1016/j.lwt.2004.07.012

[7] Pooley R, Peterson L. Mechanisms of microbial susceptibility and resistance to antimicrobial agents. In: Shulman ST, Phair JP, Peterson LR, John R, Warren, editors. The biologic and clinical basis of Infectious diseases. 5th ed. J,R.; Philadelphia: W.B. Saunders Company; 1997. p. 550.

[8] Somova LI, Shode FO, Ramnanan P, Nadar A. Antihypertensive, antiatherosclerotic and antioxidant activity of tripenoids isolated from Olea europaea. Subspecies Africana Leaves J Ethnopharmacol. 2003;84:299–305. 12648829 10.1016/S0378-8741(02)00332-X

[9] Benavente-Garcia O, Castillo J, Lorente J, Ortuno A, Del-Rio JA. Antioxidant activity of phenolics from Olea europaea L. leaves. Food Chem. 2000;49(4):2480–5.

[10] Tutour BL, Guedon D. Antioxidative activities of Olea Europea leaves and related phenolic compounds. Phytochemistry. 1992;31(4):1173–8. 10.1016/0031-9422(92)80255-D

[11] Visioli F, Galli C. Olives and their production waste products as sources of bioactive compounds. Curr Top Nutraceutical Res. 2003;1:85–8.

[12] Owen RW, Giacosa G, Hull WE, Haubner R, Wurtele G, Spiegelhalder B, Bartsch H. Olive oil consumption and health: the possible role of antioxidants. Lancet Oncol. 2000;1(2):107–12. 11905662 10.1016/S1470-2045(00)00015-2

[13] Basch E, Ulbricht V, Hammerness P, Bevins A, Sollars D. Thyme (Thymus vulgarisl) thymol. J Herb Pharmacother. 2004;4:63–78. 15273078

[14] Imelouane B, Hassa A, Wathelet IP, Ankit M, Khedid K, El-Bachiri AA. Chemical composition and antimicrobial activity of essential oil of thyme (Thymus Vulgaris) from eastern Morocco. Int J Agric Biol. 2009;11:205–8.

[15] Grigore A, Paraschiv I, Mihul A, Corina B, Draghici EM, Ichim M. Chemical composition and antioxidant activity of Thymus vulgaris L. volatile oil obtained by two different methods. Rom Biotechnol Lett. 2010;15:5436–43.

[16] Al Maqtari M, Alghalibi S, Alhamzy E. Chemical composition and antimicrobial activity of essential oil of Thymus vulgaris from Yemen. Turk J Biochem. 2011;36:342–9.

[17] Aliabadi MA, Darsanaki RK, Rokhi ML, Nourbakhsh M, Raeisi G. Antimicrobial activity of olive leaf aqueous extract. Ann Biol Res. 2012;3:4189–91.

[18] Al-Rimawi F, Sbeih M, Amayreh M, Rahhal B, Mudalal S. Evaluation of the effectiveness of natural extract as a substituent for synthetic preservatives and antioxidants in pharmaceutical preparations. Saudi Pharm J. 2024;32(5). 10.1016/j.jsps.2024.102014. 10.1016/j.jsps.2024.102014 PMC10975503 38550330

[19] Zorić N, Kosalec I. The Antimicrobial activities of Oleuropein and Hydroxytyrosol. In: Rai M, Kosalec I, editors. Promising antimicrobials from Natural products. Cham: Springer; 2022. 10.1007/978-3-030-83504-0_5.

[20] Salma NM. Antibacterial activity of Olive (Olea europaea) leaves and Arugula (Eruca sativa) seeds extract. Int J Pharmacogn Phytochem. 2015;7:307–10.

[21] Markin D, Duek L, Berdicevsky I. In vitro antimicrobial activity of olive leaves. Mycoses. 2003;46:132–1366. 12870202 10.1046/j.1439-0507.2003.00859.x

[22] Battinelli L, Daniele C, Cristiani G, Mazzanti G. In vitro antifungal and anti-elastase activity of some aliphatic aldehydes from Olea europaea L. fruit. Phytomedicine. 2006;13:558–63. 16920510 10.1016/j.phymed.2005.09.009

[23] KarygianniL, Cecere M, Skaltsounis AL, Argyropoulou A, Hellwig E, Aligiannis N, Wittmer A, Al-Ahmad A. High-level Antimicrobial Efficacy of Representative Mediterranean Natural Plant extracts against oral microorganisms. Biomed Res Int 2014; Article ID 839019, 8 pages. 10.1155/2014/839019 PMC4098616 25054150

[24] Gumgumjee NM, Hajar AS. Antimicrobial activities and phytochemical properties of Saudi oleaeuropaea subsp cuspidate. Life Sci J. 2014;11:232–7.

[25] Gokmen M, Kara R, Akkaya L, Torlak E, Onen A. Evaluation of antimicrobial activity in olive (olea europaea) leaf extract. Am J Microbiol. 2014;5:37–40.

[26] Korukluoglu M, Sahan Y, Yigit A. Antifungal properties of olive leaf extracts and their phenolic compounds. J Food Saf. 2008;28:76–87. 10.1111/j.1745-4565.2007.00096.x

[27] Pereira AP, Ferreira ICFR, Marcelino F, Valentao P, Andrade PB, Seabra R, Estevinho L, Bento A, Pereira JA. Phenolic compounds and Antimicrobial Activity of Olive (Olea europaea L. Cv Cobrancosa) Leaves Molecules. 2007;12:1153–62. 17873849 10.3390/12051153 PMC6149345

[28] Erdohan O, Turhan KN. Olive leaf extract and usage for development of antimicrobial food packaging, Science against microbial pathogens: communicating current research and technological advances. Publisher: Formatex, ; 2012. pp. 1094–101. Department of Food Engineering, University of Mersin, 33343, Çiftlikköy, Mersin, Turkey.

[29] Ilias F, Kholkhal W, Gaouar N, Bekhechi C, Bekkara FA. Antibacterial and antifungal activities of olive (Olea europaea L.) from Algeria. J Microbiol Biotechnol res. 2011;1:69–73.

[30] Hussain A, Qarshi QA, Liaqat R, Akhtar S, Aziz I, Ullah I, Shinwari ZK. Antimicrobial potential of leaf and fruit extracts and oils of wild and cultivate dedible olive. Pak J Bot. 2014;46:1463–8.

[31] Dada AA, Ifesan BOT, Fashakin JF. Antimicrobial and antioxidant properties of selected local spices used in Kunun Beverage in Nigeria. Acta Sci Pol Technol Aliment. 2013;12:373–8.

[32] Antunes BDF, Otero DM, Oliveira FM, Jacques AC, Gandra EA, Zambiazi RC. Antioxidant and antimicrobial activity of olive trees cultivated in the Campanha Gaúcha region. Braz J Dev. 2020;6:21791–805. 10.34117/bjdv6n4-374

[33] Liu Y, McKeever LC, Malik NSA. Assessment of the antimicrobial activity of Olive Leaf Extract against Foodborne bacterial pathogens. Front Microbiol. 2017;8:113. 28210244 10.3389/fmicb.2017.00113 PMC5288333

[34] Rafiei Z, Jafari SM, Alami M, Khomeiri M. Evaluation of antimicrobial activity of olive leaf extracts by ELISA method. IJMAPR. 2012;28:280–92.

[35] Korukluoglu M, Sahan Y, Yigit A, Karakas R. Antifungal activity of olive leaf (Olea Europaea L.) extracts from the Trilye Region of Turkey. Ann Microbiol. 2006;56:359–62. 10.1007/BF03175032

[36] Korukluoglu M, Sahan Y, Yigit A, Ozer ET, Gucer S. Antibacterial activity and chemical constitution so Olea europaea L. leaf extracts. J Food Process Preserv. 2010;34:383–96. 10.1111/j.1745-4549.2008.00318.x

[37] Borjan D, Leitgeb M, Knez Z, Hrncic MK. Microbiological and antioxidant activity of Phenolic compounds in Olive Leaf Extract. Molecules. 2020;25:5946. 33334001 10.3390/molecules25245946 PMC7765412

[38] Simat V, Skroza D, Tabanelli G, Cagalj, Pasini F, Gomez-Caravaca AM, Fernandez-Fernandez C, Sternisa M, Mozina SS, Ozogul Y, Mekinic IG. Antioxidant and antimicrobial activity of Hydroethanolic Leaf extracts from six Mediterranean Olive cultivars. Antioxidants. 2022;11(9):1656. 36139730 10.3390/antiox11091656 PMC9495989

[39] Nasrollahi Z, Abolhasannezhad M. Evaluation of the antifungal activity of olive leaf aqueous extracts against *Candida albicans* PTCC-5027. Cur Med Mycol. 2015;1(4):37–9. 10.18869/acadpub.cmm.1.4.37 PMC5490280 28681003

[40] Giacometti J, Milovanovic S, Momcilovic MJ, Bubonja-Sonje M. Evaluation of antioxidant activity of olive leaf extract obtained by ultrasound-assisted extraction and their antimicrobial activity against bacterial pathogens from food. Int J Food Sci Technol. 2021;56:4843–50. 10.1111/ijfs.15319

[41] Guo L, Gong S, Wang Y, Sun Q, Duo K, Fei P. Antibacterial Activity of Olive Oil Polyphenol Extract against Salmonella Typhimurium and *Staphylococcus aureus*: possible mechanisms. Foodborne Pathog Dis. 2020;17(6):396–403. 31755743 10.1089/fpd.2019.2713

[42] Marino M, Bersani C, Comi G. Antimicrobial activity of the essential oils of Thymus vulgaris L. measured using a bioimpedometric method. J Food Prot. 1999;62(9):1017–23. 10492476 10.4315/0362-028X-62.9.1017

[43] Boskovica M, Zdravkovic N, Ivanovic J, Janjic J, Djordjevic J, Starcevic M, Baltic MZ. Antimicrobial activity of Thyme (Tymus Vulgaris) and oregano (Origanum vulgare) essential oils against some food-borne microorganisms. Procedia Food Sci. 2015;5:18–21. 10.1016/j.profoo.2015.09.005

[44] Cetin B, Cakmakci S, Cakmaci R. The investigation of antimicrobial activity of thyme and oregano essential oils. Turk J Agric for. 2011;35(2):145–54.

[45] Gavaric N, Mozina SS, Kladar N, Bozin B. Chemical profile, antioxidant and antibacterial activity of thyme and oregano essential oils, thymol and carvacrol and their possible synergism. J Essent Oil Bear Plants. 2015;18(4):1013–21. 10.1080/0972060X.2014.971069

